# Supplementary material for: Evaluation of the Relationship between Lower Limb Hypermobility and Ankle Muscle Strength in a Paediatric Population: Protocol for a Cross Sectional Study
Source: Int J Environ Res Public Health. 2022 Jun 14;19(12):7264. doi: 10.3390/ijerph19127264 (PMC9223762; doi:10.3390/ijerph19127264)
Supplement: Supplementary file 1 [file ijerph-19-07264-s001.zip › ijerph-1663232-supplementary.pdf]

Appendix SA

San Francisco de ASIS

SDAD.COOP. DE ENSEÑANZA
SAN FRANCISCO DE ASIS DE LORCA
COLEGIO CONCERTADO

C/ NOGALTE, Nº 3, 30800 LORCA (MURCIA)
TLFNO: 968 466 206
www.colegiosanfranciscolorca.es

CONSENTIMIENTO INFORMADO

Estimados padres, madres y tutores/as,

Como en años anteriores, nos ponemos en contacto con ustedes para darles a conocer las actividades que se van a desarrollar en el Colegio San Francisco de Asís, dentro del Programa de Atención Podológica en la Población Escolar (PAPPE), que hemos desarrollado desde la Facultad de Ciencias de la Salud de la Universidad de Málaga y Murcia.

Con el programa PAPPE se pretende detectar alteraciones podológicas en los niños y niñas, así como su relación con el sobrepeso y la obesidad en edades tempranas.
Las actividades serán llevadas a cabo por podólogos de la Clínica Verónica Díaz, Lorca. Esta actividad es estrictamente observacional y, por tanto, no entraña ningún riesgo para el alumno/a del Centro, pero permite, en caso de detección de alguna anomalía, ofertar consejo sanitario a los padres.

Los datos obtenidos se incluirán en el registro clínico de atención podológica del Departamento de Enfermería y Podología, respetando la identidad de los participantes conforme a las normas éticas y de protección de datos pertinentes. Esta información se utilizará exclusivamente con finalidad asistencial en el caso de que lo requiriesen los afectados y para la docencia e investigación en procesos podológicos infantiles. Al finalizar el estudio, cada una de las familias recibirá un informe para conocer las características podológicas de sus hijos.
Esperamos que valoren positivamente la realización de esta actividad y colaboren en este proyecto, quedando a su disposición para cualquier información relacionada con la salud de los pies de sus hijos.

Atentamente, le enviamos un cordial saludo.
Si desea que le realicen el estudio a su hijo, rellena los datos y entrega esta hoja a su tutor.
D./Dña.. con DNI............................
Padre, Madre o Tutor del alumno/a..
del Colegio San Francisco de Asís, Lorca.

Autorizo la realización de la exploración podológica a mi hijo/a durante el periodo de primaria.
Firma,

Appendix SB

Name **Nº=**

Age:

- **Anthropometry**

Weight Height

- **FPI**

	FACTOR	PLANE	SCORE 1 Date_____ Comment_____		SCORE 2 Date_____ Comment_____	
			Left -2 to +2	Right -2 to +2	Left -2 to +2	Right -2 to +2
Rearfoot	Talar head palpation	Transverse				
	Curves above and below the lateral malleolus	Frontal/ transverse				
	Inversion/eversion of the calcaneus	Frontal				
Forefoot	Prominence in the region of the TNJ	Transverse				
	Congruence of the medial longitudinal arch	Sagittal				
	Abd/adduction forefoot on rearfoot	Transverse				
	TOTAL					

LUNGE TEST

Lunge test Right: __________ ____________

Lunge test Left: ____________ ______________

- **TEST RCSP**

Left _______ _________

Right _________ __________

- **Beighton scale**

Test	Asignación de Puntaje
1) Passive dorsiflexion of the little fingers beyond 90°	Right:<br>Left:
2) Passive approximation of the thumb to the ventral aspect of the forearm.	Right:<br>Left:
3) Hyperextension of the elbow joint beyond 10°	Right:<br>Left:
4) Hyperextension of the knee joint beyond 10°	Right:<br>Left:
5) Placing the palms of the hands on the floor while keeping the knees in full extension	
score out of 9	

- **Lower Limb Assessment Score (LLAS)**

	LEFT		RIGHT	
Hip flexion	YES	NO	YES	NO
Hip abduction	YES	NO	YES	NO
Knee hyperextension	YES	NO	YES	NO
Knee anterior drawer test	YES	NO	YES	NO
Knee rotation	YES	NO	YES	NO
Ankle dorsiflexion	YES	NO	YES	NO

Ankle anterior drawer test	YES	NO	YES	NO
Subtalar inversion	YES	NO	YES	NO
Midtarsal inversion	YES	NO	YES	NO
Midtarsal ab/adduction and dorsi/plantarflexion	YES	NO	YES	NO
Metatarsophalangeal movement	YES	NO	YES	NO
Excessive subtalar joint pronation (weight-bearing)	YES	NO	YES	NO
	**Total**:<br>score out of 12			

Appendix SC

- **<u>Ankle Strength</u>**
    1. Right inversion strength
    2. Right inversion strength
    3. Right inversion strength

    1. Left inversion strength
    2. Left inversion strength
    3. Left inversion strength

    1. Right eversion strength
    2. Right eversion strength
    3. Right eversion strength

    1. Left eversion strength
    2. Left eversion strength
    3. Left eversion strength

    1. Right plantarflexion strength
    2. Right plantarflexion strength
    3. Right plantarflexion strength

    1. Left plantarflexion strength
    2. Left plantarflexion strength
    3. Left plantarflexion strength

    1. Right dorsiflexion strength
    2. Right dorsiflexion strength
    3. Right dorsiflexion strength

    1. Left dorsiflexion strength
    2. Left dorsiflexion strength
    3. Left dorsiflexion strength

- TRF: __________ ____________ _____________
- 5-JT: __________ ____________ _____________

Appendix SD

- 6MWT: _________________ meters
- 10MWT: _________________ seconds
- 10MRT: __________________ seconds
